# JNK1 activation predicts the prognostic outcome of the human hepatocellular carcinoma

**DOI:** 10.1186/1476-4598-8-64

**Published:** 2009-08-17

**Authors:** Qingshan Chang, Jianguo Chen, Kevin J Beezhold, Vince Castranova, Xianglin Shi, Fei Chen

**Affiliations:** 1 Graduate Center for Toxicology, University of Kentucky, Lexington, KY40536, USA; 2 Pathology and Physiology Research Branch, The Health Effects Laboratory Division, National Institute for Occupational Safety and Health, Morgantown, WV 26505, USA; 3 Qidong Liver Cancer Institute, Jiangsu Province, Qidong 226200, PR China

## Abstract

**Background:**

Hepatocellular carcinoma (HCC) is one of the most common cancers worldwide with an extremely poor prognosis. The classification of HCC based on the molecular signature is not well-established.

**Results:**

In the present study, we reported HCC signature genes based on the JNK1 activation status in 31 HCC specimens relative to the matched distal noncancerous liver tissue from 31 patients. The HCCs with high JNK1 (H-JNK1) and low JNK1 (L-JNK1) were sub-grouped. Two different signature gene sets for both H-JNK1 and L-JNK1 HCC were identified through gene expression profiling. A striking overlap of signature genes was observed between the H-JNK1 HCC and the hepatoblastoma or hepatoblastoma-type HCC. Many established biomarkers for hepatic progenitor cells were over-expressed in H-JNK1 HCC, including AFP, TACSTD1, KRT19, KRT7, THY1, and PROM1. In addition, the majority of the most up-regulated genes were those associated with metastasis and earlier recurrence, whereas the genes for normal liver function were substantially down-regulated in H-JNK1 HCC tissue. A Kaplan-Meier plot demonstrated that the survival of the patients with H-JNK1 HCC was severely impaired.

**Conclusion:**

Accordingly, we believe that the H-JNK1 HCC may originate from hepatic progenitor cells and is associated with poorer prognosis. The status of JNK1 activation in HCC tissue, thus, might be a new biomarker for HCC prognosis and therapeutic targeting.

## Background

Hepatocellular carcinoma (HCC) has plagued populations in Far East Asia, South Asia and Sub-Saharan Africa for several decades where the prevalence of hepatitis B viral (HBV) infection and aflatoxin exposure is high[1,2]. A sharp increase in HCC incidence in North America, Western Europe and Japan has been noted in recent years due to hepatitis C viral (HCV) infection, alcohol abuse and non-alcoholic fatty liver disease [3-6]. HCC is the fourth most common neoplasm and the third most common cause of cancer-related death worldwide. Age-adjusted HCC incidence rates vary from 2 per 100,000 population in North America to 80 per 100,000 population in China. Since most HCC patients are diagnosed when the tumors are in an advanced stage and the majority of HCCs develop in the context of chronic liver cirrhosis, curative therapy is not available yet[7,8]. The overall 5-year survival rate is lower than 5% [4]. Accordingly, a new understanding of the molecular mechanisms controlling the development and progression of HCC is urgently needed for the efficient treatment of this deadly disease.

A major goal in current HCC research is to define gene signatures that drive initiation, maintenance and progression of the malignant tumors, which is anticipated to be helpful in classifying tumor stages and predicting prognostic outcomes, such as metastasis, patient survival rate and recurrence of the tumors after resection. It is also highly desirable to use the gene signature to design targeted therapies or the so-called personalized medicine [1]. However, amid this euphoria, the HCC signature genes identified thus far vary considerably, depending on a number of etiological and pathological features, including viral infection, inflammation, cirrhosis, necrosis, fibrosis, vascular invasion, tumor cell origination, and environmental exposure. The situation is further complicated by the byzantine complexity of algorithms for data analysis and the use of different platforms for gene profiling [9-12]. These factors might explain why so many reports concerning HCC signature genes have resulted in therapeutic dead ends.

Accumulating evidence suggests that activation of the protein kinases provides growth advantage to cells during malignant transformation and tumorigenesis. Recently, we[13,14] and Hui et al.[15] simultaneously demonstrated critical role of JNK1 activation in the pathogenesis of human HCC and/or a mouse model of chemical carcinogen-induced HCC. Both studies observed overactivation of JNK1 in about 55% and 56% human HCC relative to the noncancerous liver tissue, respectively[13,15]. We have shown an association between JNK1 activation and overexpression of the genes for cell growth, histone methylation, and the downregulation of the genes for cell differentiation, antioxidant defense and drug or lipid metabolism[13]. By re-analyzing and comparing our gene profiling data with the signature genes for HCC with a poor prognosis and hepatoblastoma (HB) reported recently[9,16], we found a striking overlap of the gene expression pattern in high JNK1 (H-JNK1) HCC tissue with the signature genes for HCC with a poor prognosis and HB, respectively. Assessment of the patient survival data indicates that the overall survival of the patients with H-JNK1 HCC is substantially impaired. JNK1, rather than JNK2, has been implicated in development of steatohepatitis[17,18] and carcinogen-induced mouse HCC[19]. It is very likely, thus, that JNK1 is a critical contributor to the progression of human HCC, which can serve as a potential target for new therapies.

## Results

### Gene profiling between HCC and the noncancerous liver tissue

HCC samples along with the case-matched distal noncancerous liver tissue (ANC) were collected from 31 patients (Table 1) during year 2005 and 2006 and gene profiling using Affymetrix HG-U133 plus 2 microarrays was performed as reported previously[13]. The data were re-normalized using the GCRMA method and FDR correction with a p-value of ≤ 0.05. The probe sets with "absence" call on the array were removed before normalization. A total of 852 genes showed differential expression between HCC and ANC samples (Table 2 and Additional file 1). The up-regulated 515 genes and down-regulated 337 genes from the FDR-corrected data covered 41% and 72% of the up- and down-regulated genes, respectively, listed in the data generated by Welch's T-test with Bonferroni correction and p ≤ 0.001[13]. As reported previously, genes for DNA synthesis, cell cycle and ubiquitination, including RRM2, CCNB1, AURKA, NUSAP1, UBE2S, UBE2T, etc. were over-expressed; whereas genes for cell death and liver function, such as Egr1, MT1F and p450 family members, were under-expressed in HCC relative to the ANC tissue.

**Table 1:** Clinical information for the H-JNK1 and L-JNK1 HCC patients

	H-JNK1 n = 17	L-JNK1 n = 14	p – value
Sex (M/F)	13/4	10/4	0.5616
Age, y (range)	55.6 (32–70)	52.3 (42–69)	0.4110
HBV	13	9	0.4903
HCV	nd*	nd	
Cirrhosis	5	9	0.1936
Encapsulation	5	9	0.1936
AFP (+/-)	12/5	9/5	0.5486
> 200	9	5	0.3971
> 400	8	3	0.2507
Tumor grade (II/III)	7/10	9/5	0.3401
Tumor diameter (cm)	7.3 ± 2.8	5.0 ± 2.7	**0.0270**
Survival (months)	21.7 ± 19**	39.1 ± 30	0.073
Male	14 ± 14	42 ± 30	**0.0120**
Female	43 ± 14	28 ± 34	0.459

*nd: not determined

**data for 2 out of 31 patients were not available due to loss of contact.

**Table 2:** The 10 most up- and down-regulated genes in HCC tissue vs ANC tissue

Probe ID	Fold	FDR	Gene	Description
209773_s_at	42.92361	0.049469	RRM2	ribonucleotide reductase M2
214710_s_at	31.22006	0.045816	CCNB1	cyclin B1
208079_s_at	22.2925	0.045693	AURKA	aurora kinase A
218039_at	14.23746	0.045693	NUSAP1	nucleolar/spindle protein 1
202779_s_at	14.00133	0.045816	UBE2S	ubiquitin-conjugating E2S
225687_at	13.5030	0.036302	FAM83D	family with similarity 83 D
218883_s_at	13.12003	0.046408	MLF1IP	MLF1 interacting protein
219787_s_at	12.05556	0.049035	ECT2	epithelial transforming 2
209218_at	11.85705	0.048765	SQLE	squalene epoxidase
223229_at	11.59042	0.045816	UBE2T	ubiquitin-conjugating E2T
				
223699_at	-52.7765	0.028925	CNDP1	carnosine dipeptidase 1
202992_at	-54.1709	0.033434	C7	complement component 7
206727_at	-59.2	0.045816	C9	complement component 9
207102_at	-61.2122	0.048508	AKR1D1	aldo-keto reductase 1D1
230478_at	-73.8474	0.02547	OIT3	oncoprotein induced transcript 3
205866_at	-75.0982	0.014497	FCN3	ficolin 3
206354_at	-96.8498	0.028696	SLCO1B3	solute carrier organic transporter
220491_at	-97.3833	0.028696	HAMP	hepcidin antimicrobial peptide
1554459_s_at	-98.797	0.045816	CFHR3	complement factor H-related 3
207874_s_at	-112.08	0.046995	CFHR4	complement factor H-related 4

### JNK1 activation in HCC

**Figure 1:**
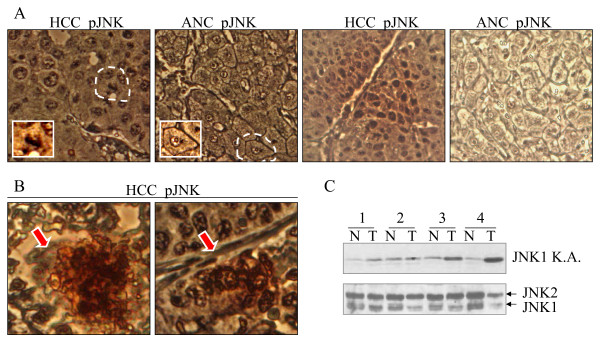
**Activation of JNK is enhanced in HCC samples**. (A). Tissue microarray with paired human HCC and the adjacent non-cancerous tissue (ANC) was stained with pJNK antibody. The inserted panels show a typical immunostaining pattern of pJNK in a single HCC cell and non-cancerous cell, respectively; (B). Enhanced pJNK staining in the small foci of necrosis was indicated by the red arrows in HCC tissue. (C). JNK1 specific kinase activity assay in 4 HCC tissue samples (T) paired with the non-cancerous liver tissues (N).

We had previously shown activation of JNK1 in about 55% of human HCC samples as compared with the case-matched ANC samples through immunoblotting[13]. To validate these observations again, we also examined JNK activation status in some additional HCC tissue samples paired with ANC tissue in tissue microarray through immunohistochemistry (IHC) analysis (Fig. 1). Since an antibody specific for phosphorylated-JNK1 (pJNK1) was unavailable, we used an antibody against both pJNK1 and pJNK2. Despite the fact that both HCC and ANC exhibited a similar weak background signal in IHC, possibly due to pJNK2 as demonstrated in our previous Westernblotting[13], 24 out of 52 HCC samples (46%) in the tissue microarray slides showed a notable enhancement of pJNK signal as compared with the ANC tissue (representative images in Fig. 1A). Of note, the enhancement of JNK activation as implicated in the phosphorylation of the JNK largely occurred in the nuclei of the hepatocytes (top panels in Fig. 1A) and was not uniformly distributed in the whole HCC tissue in the majority of tissue slides, but rather, clustered in certain limited areas, especially in those areas featured with small foci of necrosis (Fig. 1B). The JNK1 activation was further validated by a JNK1 specific kinase activity assay in additional 4 HCC samples paired with the ANC tissues (Fig. 1C).

### JNK1-associated HCC signature genes

**Figure 2:**
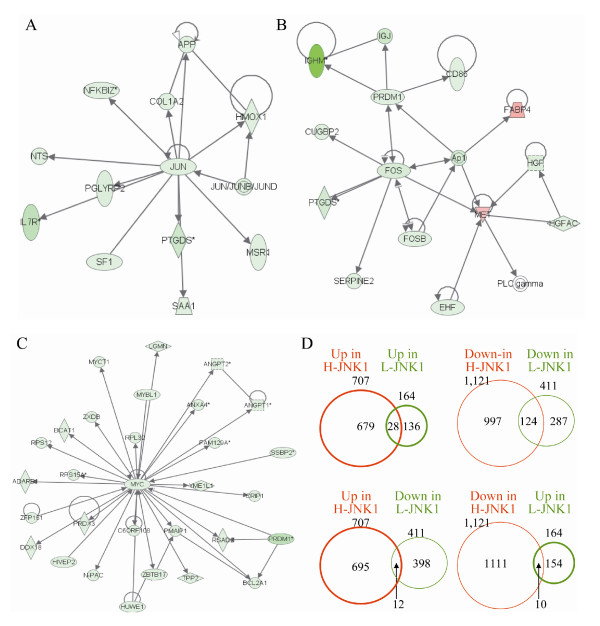
**The Jun, Fos and Myc signaling pathways were down-regulated in the low JNK1 (L-JNK1) HCC tissue**. Genes associated with the Jun signaling (A), Fos signaling (B) and Myc (C) signaling pathways were down-regulated in L-JNK1 HCC tissue. (D) Cross-coverage of the genes between H-JNK1 and L-JNK1 HCC samples were measured by Venn Diagrams.

Based on the JNK1 activation status in HCC relative to the ANC tissue, we further classified gene expression profiles associated with H-JNK1 HCC and L-JNK1 HCC, respectively. To gain strong biological and statistical significance, the genes with a larger than 5-fold differential ratio between HCC and ANC samples were selected for further analysis. A list of 1,828 genes was generated for the H-JNK1 HCC, among which 707 genes were up-regulated and 1,121 genes were down-regulated (Additional file 2). In L-JNK1 HCC samples, 164 genes were up-regulated and 411 genes were down-regulated (Additional file 3). The lower JNK1 activity in this group of HCC samples was supported by the down-regulation of the Jun, Fos and Myc signaling pathways in an interactive network analysis (Figs. 2A, B &2C). By comparing the up- and down-regulated genes in these two groups, only 28 genes were commonly up-regulated (Fig. 2D and Additional file 4) and 124 genes were commonly down-regulated between them (Fig. 2D and Additional file 5). Furthermore, 12 up-regulated genes in H-JNK1 HCC samples were down-regulated in the L-JNK1 HCC samples (Fig. 2D and Additional file 6) and 10 down-regulated genes in the H-JNK1 HCC samples were up-regulated in the L-JNK1 HCC samples (Fig. 2D and Additional file 6). Accordingly, the vast majority of the genes identified are truly associated with the JNK1 activation status in HCC samples.

**Figure 3:**
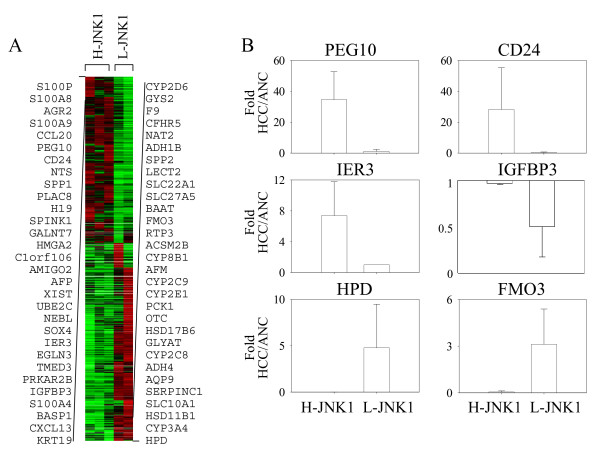
**Signature genes of the H-JNK1 HCC samples**. (A). The 30 most up-regulated and the 30 most down-regulated genes were generated from direct comparison of the gene profiling between H-JNK1 HCCs and L-JNK1 HCC samples. (B). Relative expression levels of PEG10, CD24, IER3, IGFBP3, HPD, and FMO3 in H-JNK1 HCC and L-JNK1 HCC samples.

**Figure 4:**
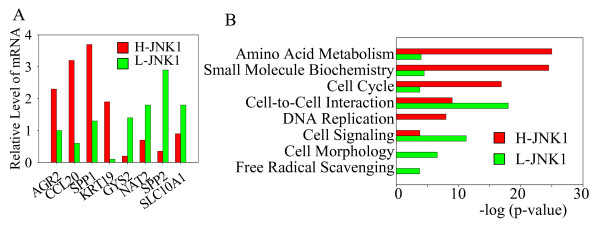
**Comparison of genes and pathways between H- and L-JNK1 HCCs through real-time PCR and pathway assay**. (A). Quantitative real-time PCR of the selected genes showing differential expression between H- and L-JNK1 HCC. (B). Different expression levels of the genes associated with these indicated interactive pathways were determined by comparing pathway molecules between H-JNK1 HCC and L-JNK1 HCC samples through Ingenuity Pathways Analysis.

To determine potential signature genes defining the H-JNK1 and L-JNK1 HCC tissue, a new gene list was generated by direct comparing the genes in H-JNK1 HCC to the L-JNK1 HCC samples. This comparison showed that 927 genes were over-expressed and 1,057 genes were under-expressed in H-JNK1 HCC samples relative to the L-JNK1 HCC samples with a differential ratio large than 5 fold (Additional file 7). The 30 most up-regulated and the 30 most down-regulated genes in H-JNK1 HCC tissue are shown in Fig. 3A. Some of the top 30 genes are associated with the liver progenitor cells and the imprinted genes, such as PEG10, CD24, H19, and KRT19. Several genes representing the normal liver function, such as CYP family members, NAT2, ADH1B, and ADH4, are among the 30 most down-regulated genes. The relative expression levels of PEG10, CD24, IER3, IGFBP3, HPD and FMO3 in H-JNK1 and L-JNK1 HCC tissue are shown in Fig. 3B. It should be noted that although both H-JNK1 and L-JNK1 HCCs samples showed decreased expression of IGFBP3 relative to the matched ANC samples as reported by others[20], the IGFBP3 level in L-JNK1 HCC tissue is much lower than that in H-JNK1 HCC tissue (Fig. 3B). The differential expression levels of some of these genes between H-JNK1 and L-JNK1 HCC were additionally validated by quantitative real-time PCR (Fig. 4A).

An additional functional comparison between these two groups indicates that the expression of the genes for amino acid metabolism, small molecule biochemistry, cell cycle, and DNA replication was overexpressed in the H-JNK1 HCC tissue relative to the L-JNK1 HCC tissue (Fig. 4B). In contrast, the genes for cell-to-cell interaction, cell signaling, cell morphology, and free radical scavenging were up-regulated in the L-JNK1 HCC tissue relative to the H-JNK1 HCC tissue (Fig. 4B).

### Biological networks in H-JNK1 HCC vs L-JNK1 HCC

**Figure 5:**
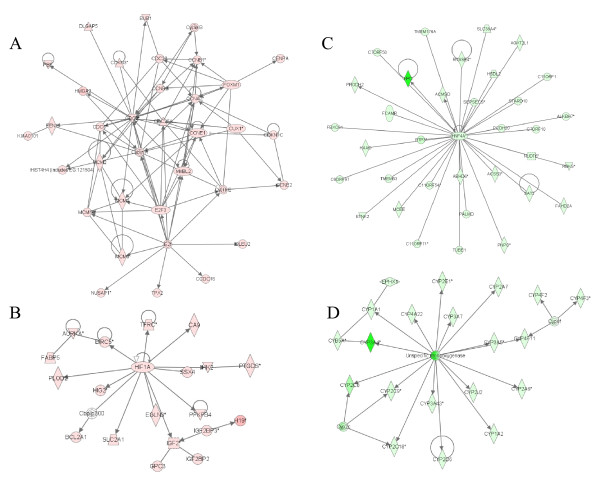
**Major signaling pathways were altered in H-JNK1 HCC tissue**. Genes for cell cycle pathway (A) and HIF-1 signaling (B) were up-regulated in H-JNK1 HCC tissue as determined by Ingenuity Pathways Analysis. Genes involved in HNF4A transcription network (C) and for the p450 family members (D) were down-regulated in H-JNK1 HCC tissue.

To gain insights into the JNK1 activation and HCC pathogenesis, we carried out additional interactive gene network analysis using the Ingenuity Pathways Analysis software for those differentially regulated genes between H-JNK1 and L-JNK1 HCC samples with a ratio larger than 5 fold. Among the 927 up-regulated genes in H-JNK1 HCC samples relative to the L-JNK1 HCC samples, many genes can be mapped to the top five interaction networks for cell growth, cell cycle, cell death, cellular assembly, and DNA replication (Fig. 5A and Additional file 8). Intriguingly, HIF1A and many of its targeting molecules were also up-regulated, further supporting the highly proliferative nature of the H-JNK1 HCC tissue (Fig. 5B). The top interactive networks for the down-regulated genes in the H-JNK1 HCC samples relative to the L-JNK1 HCC samples are those involved in hepatic transcription factor HNF4A network and metabolisms for lipids and drugs, such as the p450 family members (Figs. 5C and 5D; Additional file 8).

### Overlapping of the signature genes between H-JNK1 HCC tissue and HCC with poor prognosis

A number of previous studies reported potential HCC signature genes associated with metastasis, recurrence and patient survival [9,21-24]. To determine whether there is a concordance between the JNK1-related gene profiling and those previously reported HCC signature genes, we compared our data with some of these earlier reports. To simplify such comparison, we arbitrarily selected the 30 most up-regulated and the 30 most down-regulated genes, as indicated in Fig. 3A, in the H-JNK1 HCC tissue as compared with the L-JNK1 HCC tissue. Among the top 30 genes overexpressed in the H-JNK1 HCC tissue relative to the L-JNK1 HCC tissue, 23 genes had been previously implicated as signature genes either for HCC itself or for the prediction of poorer prognostic outcomes, such as impaired patient survival, intra-hepatic or extra-hepatic metastasis, and earlier recurrence of the tumor after surgical resection. These overlapping HCC signature genes include S100P [12], S100A8 [22], S100A9 [12], CCL20 [11,25], PEG10 [16], CD24 [11,16,26], NTS [9], SPP1[24], PLAC8 [27], H19 [28,29], SPINK1 [30], GALNT7 [11] HMGA2 [16], C1orf106 [12], AMIGO2[11,23], AFP [11,22], UBE2C [11,31], SOX4[11,16,32], IER3 [9], TMED3 [12], IGFBP3 [33], S100A4 [34], and KRT19 [22] (Table 3).

**Table 3:** Overlapping of H-JNK1 signature genes with other types of HCCs

Up in H-JNK/L-JNK		HB HCC^§^	Up In HB**	mTOR HCC^¶^	EpCAM HCC^†^	Down in H-JNK1/L-JNK1		HB HCC	Down In HB	mTOR HCC	EpCAM HCC
Genes	Fold		rC2/rC1*			Genes	Fold		rC2/rC1		

S100P	611				Y	CYP2D6	-112	Y	0.1		Y
S100A8	371	Y	a		Y	GYS2	-122		0.1	Y	Y
AGR2	309					F9	-141		0.1		Y
S100A9	278		a		Y	CFHR5	-148				Y
CCL20	266			Y	Y	NAT2	-151		0.2		Y
PEG10	206		14.9		Y	ADH1B	-151		0.1		
CD24	163		6.7	Y	Y	SPP2	-154		a		
NTS	143	Y			Y	LECT2	-155	Y		Y	
SPP1	126				Y	SLC22A1	-156	Y	0.1		Y
PLAC8	121		a		Y	SLC27A5	-156		0.2		Y
H19	92		qPCR		Y	BAAT	-173			Y	Y
SPINK1	78				Y	FMO3	-183	Y	0.1	Y	Y
GALNT7	63				Y	RTP3	-217				Y
HMGA2	61		5.5		Y	ACSM2B	-222				Y
C1orf106	61				Y	CYP8B1	-224	Y		Y	Y
AMIGO2	55				Y	AFM	-239		a	Y	
AFP	54		35		Y	CYP2C9	-242	Y	0.2	Y	
XIST	52					CYP2E1	-244	Y	0.2	Y	
UBE2C	51				Y	PCK1	-251	Y	0.1		Y
NEBL	49					OTC	-276	Y			Y
SOX4	48		3.0		Y	HSD17B6	-298		0.1		Y
IER3	45	Y			Y	GLYAT	-458	Y	0.2	Y	Y
EGLN3	42					CYP2C8	-525		0.0		Y
TMED3	40				Y	ADH4	-535			Y	
PRKAR2B	40				Y	AQP9	-684	Y	0.2	Y	Y
IGFBP3	39				Y	SERPINC1	-711	Y	a		Y
S100A4	39				Y	SLC10A1	-975		0.1		Y
BASP1	38		b			HSD11B1	-1040		0.1	Y	Y
CXCL13	37					CYP3A4	-1458	Y			
KRT19	34	Y	10.5		Y	HPD	-1864	Y	0.2	Y	

A: Up or down in later stage HB; b: Up or down in early stage HB; *: ratio of rC2/rC1; Y: covered by the indicated studies; ^§^HB HCC, ref.[22]; **HB: ref. [16]; ^¶^mTOR HCC: ref. [11]; ^†^EpCAM HCC: ref. [12].

Fifteen out of the 30 most down-regulated genes in the H-JNK1 HCC tissue were covered by those down-regulated genes in the subclass A HCC reported by Lee et al[9]. The subclass A HCC had been later confirmed as the HCCs derived from the hepatic progenitor cells with poorer survival[22]. These genes include HPD, CYP3A4, SERPINC1, AQP9, GLYAT, OTC, PCK1, CYP2E1, CYP2C9, CYP8B1, FMO3, BAAT, SLC27A5, SLC22A1, and CYP2D6 (Table 3). In addition, 9 genes, including GYS2 [11], F9 [12,35], NAT2 [11,36,37], ADH1B [26], LECT2 [11,38,39], AFM [11], ADH4 [11,21], SLC10A1 [40], and HSD11B1 [11], in this down-regulated gene list that were not covered in the down-regulated genes of Lee et al.[9], had been recently or previously reported to be substantially decreased in HCC with poorer prognosis.

### H-JNK1 HCC with features of liver progenitor cells and hepatoblastoma (HB)

The similarity between HCCs with higher JNK1 activation and the HCCs with characteristics of hepatic progenitor cells (Fig. 3A) suggests to us that the H-JNK1 HCCs possibly have features of HB. The signature genes for HB has been recently reported by Cairo et al [16]. By comparing the signature genes of HB with that of H-JNK1 HCC tissue, a striking similarity between the genes in robust Cluster 2 (rC2) HB and the genes associated with the JNK1 activation status was noted. For the top 400 increased and greatest 100 decreased genes in rC2 HB (Supplemantary table S7 of Cairo et al [16]), 90% and 94% of these genes are presented among the most increased and decreased expressed genes in the H-JNK1 HCC tissue, respectively. Vice versa, 11 out of the 30 most up-regulated genes and 20 out of the 30 most down-regulated genes in the H-JNK1 HCC tissue are represented by the up- and down-regulated genes in the HB or rC2 HB, respectively (Table 3). A significant agreement of the 16 signature genes differentiating rC2 and rC1 HB (see Cairo et al [16]) and the signature genes for the H-JNK1 HCC tissue was evident. The listed up- and down-regulated 16 signature genes in rC2 HB are fully overlap with the up- and down-regulated genes in H-JNK1 HCCs (Additional file 9).

We also noted that similar to the rC2 HB, several liver progenitor markers, such as AFP [16], TACSTD1 [41], KRT19 [16,22], KRT7 [22], PROM1 (CD133) [41,42], THY1 (CD90) [42] and VIM [22], are significantly up-regulated in the H-JNK1 HCC samples relative to the L-JNK1 HCC samples (Additional file 10). A characteristic feature of the HB and hepatic progenitor cells is the enhanced expression of the imprinted genes, including H19, IGF2, DLK1, PEG3, PEG10, MEG3, SGCE, and NDN [16]. Indeed, all of these mentioned imprinted genes are up-regulated in the H-JNK1 HCC samples (Additional file 10).

### Compromised survival of the patients with H-JNK1 HCCs

**Figure 6:**
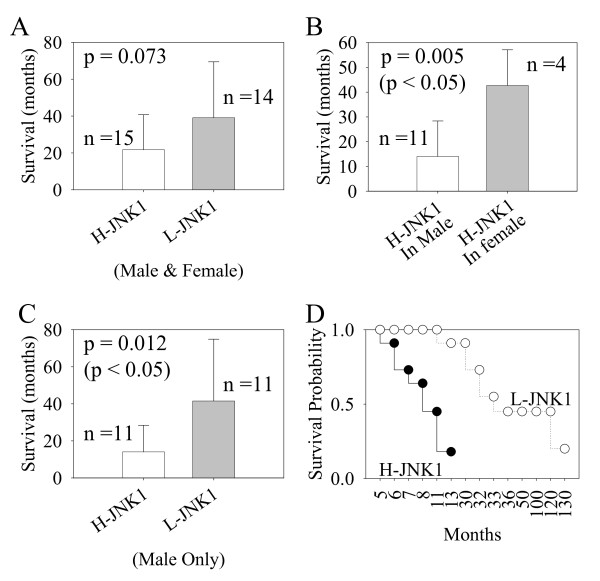
**The survival of the patients with H-JNK1 HCC was compromised**. (A). Average survival (months) of the patients (male and female) with H-JNK1 and L-JNK1 HCCs; (B). Sex discrepancy of the patients with H-JNK1 HCC. Male patients with H-JNK1 HCC have a significant shorter survival times than the female patients with H-JNK1 HCC; (C). The survival time of the male patients with H-JNK1 HCC is significantly shorter than the male patients with L-JNK1 HCC; (D). Survival probability of the male patients with H-JNK1 and L-JNK1 HCC was determined by a Kaplan-Meier estimation.

Both HB-type HCC and rC2 HB patients exhibited poorer survival relative to HC-type HCC and rC1 HB patients [16,22]. Because of the overall similarity in the gene expression profiling between the H-JNK1 HCC samples and HB or HB-type HCCs (Table 3), we thought that the status of JNK1 activation in HCC samples might be able to predict the prognostic outcome, especially on the patient survival. The patient survival data were collected during the follow-up monitoring after surgical resection of the tumor. Among the 31 patients, the survival data of 2 patients were not available due to living in an area beyond the surveillance radius and undisclosed details of home address. The remaining 29 patients were sub-grouped according to the JNK1 activation status in their tumors. The average survival of the patients with H-JNK1 HCC and L-JNK1 HCC were 21.7 and 39.1 months, respectively (Fig. 6A). However, such difference did not reach statistical significance. Since we noted a substantial difference in survival in male and female patients with H-JNK1 HCC (Table 1 and Fig. 6B), we assumed that the H-JNK1 HCC might impose stronger effect on survival for male patients. Thus, we excluded the survival data for the 7 female patients and analyzed the survival data again. The average survival of the male patients with H-JNK1 HCC is 14 months, which is significantly shorter than 41.5 months on average for the patients with L-JNK1 HCC (Fig. 6C). A Kaplan-Meier estimation of the overall survival probability at one year was 91% for patients with L-JNK1 HCC and 18% for patients with H-JNK1 HCC (Fig. 6D). Accordingly, these data suggest that the patient survival was severely impaired in H-JNK1 HCC, especially for male patients.

## Discussion

HCC is a heterogeneous type of tumor despite overall outcomes of poor prognosis. Depending on etiological and the accompanying pathological conditions, the survival or tumor recurrence vary considerably among HCC patients. The cellular origin of HCC may play key roles in determining the status of metastasis, intra-hepatic recurrence and patient survival. It has been debated for decades whether HCC arises exclusively by dedifferentiation of mature hepatocytes or maturation arrest of liver stem cells or progenitor cells (oval cells in rodents). Great efforts have been made in identifying bipotential hepatic progenitor cells that can differentiate into hepatocytes and cholangiocytes [43]. Markers for hepatocytes, such as AFP and albumin, and cholangiocytes, mainly KRT7 and KRT19, are expressed in the progenitor cells. Evidence supporting progenitor cell origin of HCC is largely based on the fact that many HCCs concurrently express both hepatocytic markers and cholangiocytic markers along with stem cell markers including TACSTD1 (EpCAM), PROM1 (CD133) and THY1 (CD90) [41,42]. The observation that a profound concordance of gene profiles for H-JNK1 HCC tissue with those of Lee et al.[22] on HB-type HCC tissue and Cairo et al. [16] on HB samples may support the hypothesis that H-JNK1 HCC is derived from the liver progenitor cells. Indeed, the genes for KRT7, KRT19, PROM1, AFP, as well as imprint genes are overexpressed in the H-JNK1 HCC tissue (Additional file 10).

The HB-like features of the H-JNK1 HCC tissue are additionally supported by the recent characterization of the EpCAM positive cells in human HCC samples. By classifying 235 HCC tumor specimens with gene profiling and immunohistochemistry analyses, Yamashita et al.[12] reported signature genes that were differentially expressed between EpCAM positive, stem cell-like HCC (HpSC-HCC) and the EpCAM negative mature hepatocyte-like HCC. A number of genes are commonly over-expressed between H-JNK1 HCC and the HpSC-HCC, such as S100P, S100A9, CCL20, PEG10, CD24, NTS, H19, UBE2C, TMED3, and KRT19 that were listed among the 30 most up-regulated genes in H-JNK1 HCC tissue (Table 3). Majority of the 30 most down-regulated genes, 22 genes, were also under-expressed in HpSC-HCC tissue. Interestingly, both H-JNK1 HCC and HpSC-HCC samples showed poorer prognosis in a Kaplan-Meier survival analysis.

Most recently, Villanueva et al.[11] reported signature genes for HCC samples with an aberrant mTOR signaling. About 40% of the 193 up-regulated genes in this type of HCC were found in the up-regulated gene list for H-JNK1 HCC samples. The coverage was increased to 86% if one relaxed the 5-fold cutoff to 1.5 fold and removed those uncharacterized genes (data not shown). For the down-regulated genes, more than 91% of the known genes were represented in the list of the down-regulated genes in H-JNK1 HCC samples. Vice versa, the genes for CCL20, CD24, GALNT7, AMIGO2, AFP, UBE2C, and SOX4 in the 30 most up-regulated genes in the H-JNK1 HCC samples were represented in the HCC samples with an aberrant activation of mTOR signaling (Table 3). The similarity in gene expression pattern between H-JNK1 HCC and the HCC with abnormal mTOR signaling further supports the assumption that H-JNK1 HCCs are the tumors with a highly aggressive nature and poorer prognosis.

It is worth to mention that there appears to be a gender disparity in JNK1 activation and HCC survival probability between male and female patients (Fig. 6B). A strong correlation between higher JNK1 activation and lower survival probability was noted in male, but not in female patients. Such disparity may be possibly attributed to the different levels of the sex steroids between two genders. It has long been hypothesized that certain female sex hormone, esp. estrogen, is repressive for the expression of inflammatory genes, such as IL-6, leading to tumor suppression [44]. In contrast, male sex hormone, androgen, appears to be a promoting factor for HCC [45]. It is unclear at the present whether the higher JNK1 activity or its down-stream effect in the female HCC patients is antagonized by the sex hormone. Nevertheless, the observed disparity in JNK1 activation and HCC prognosis between male and female patients is compensatory to the human epidemiological and animal tumorigenic studies demonstrating that the HCC incidence rate in male is about 3–5 times higher than that in female [8,45].

## Conclusion

In summary, we believe that the present report not only provides evidence indicating that JNK1 activation contributes to poorer HCC prognosis but also aids in molecular classification for HCC. It is conceivable that HCC samples with high JNK1 activation possess feature of hepatic progenitor cells and have similarities with HB[16], HB-type HCC[22], mTOR-altered HCC[11], and the EpCAM^+^/AFP^+ ^HCC (HpSC-HCC)[41]. In addition to this notion, complementary evidence to support JNK1 activation in a subset of HCC tissue was previously provided by Lee et al.[22] who demonstrated a strong enrichment of the AP-1 signaling, a direct down-stream target of JNK1, in HB-type HCC samples. The final goal of cancer gene profiling is to design targeted therapy and/or so-called personalized medicine. However, such goal is hard to achieve currently due to the large quantity of the cancer signature genes and the lack of a clear picture of the drug targetable genes or gene networks. The activation of the major signaling pathways, such as JNK1 and mTOR, on the other hand, is potentially targetable by relatively specific therapeutic agents. Thus, the identification of H-JNK1 HCC opens up possibilities to develop new treatment strategies targeting the intercellular signaling to delay the progression of HCC.

## Methods

### Human hepatocellular carcinoma specimens

The human HCC tissue samples were collected from patients undergoing surgical resection procedures with informed consent from the patients according to a protocol approved by the Institutional Review Board at Qidong Liver Cancer Institute, Qidong, PR China. Partial adjacent noncancerous (ANC) liver tissue with an average distance of 1.28 cm from the tumor were also collected for pathological evaluation. The tissue specimens were stored in liquid nitrogen immediately for later pathological and biological analyses. The diagnostic, clinical and pathological stages of the tumors were determined as previously reported[13]. Clinical relevant information for the HCC patients is shown in Table 1. A total of 40 HCC samples along with 40 case-matched ANC liver tissues were collected, among which 31 HCC samples and the matched ANC tissue were used in the present study. The other 9 HCC samples were excluded from this study because of combined cholangiocarcinoma or other type of tumors. For immunohistochemistry (IHC) studies, tissue microarray slides containing total of 52 HCC samples were purchased from US Biomax, Inc., (Rockville, MD), (See following).

### Western blotting, JNK1 kinase activity assay and Immunohistochemistry (IHC)

The JNK activation in both HCC tissues and non-cancerous liver tissues was determined through Western blotting using antibodies against phospho-JNK and total JNK purchased from Cell Signaling (Beverly, MA) as described previously[13]. JNK1 specific kinase activity assay was performed through immunoprecipitation using JNK1 antibody purchased from Santa Cruz Biotechnology Inc. (sc-474, Santa Cruz, CA) and the JNK kinase activity assay kit provided by Cell Signaling Technology (Beverly, MA). Tissue microarray slides (LV241 and LV801) purchased from US Biomax, Inc. (Rockville, MD) were used for IHC. LV241 and LV801 slides contained 12 and 40 HCC specimens along with the case-matched ANC tissue, respectively. The information about the histopathological and patient data is available at the manufacturer's website . The tissue slides were baked at 60°C for 30 min and then deparaffinized by xylene before IHC. For IHC, the slides were first subjected to antigen retrieval and then incubated with normal goat serum for 20 min at room temperature, followed by incubation with anti-phospho-JNK antibody (1:50 dilution). Other procedures of IHC were applied as suggested by the manufacturer. Specific antibodies for JNK1, JNK2 and phosphorylated-JNK were purchased from Santa Cruz Biotechnology (Santa Cruz, CA) or Cell Signaling Technology (Beverly, MA).

### Microarray and interactive pathway analyses

Gene expression profiling was performed according to the protocols described by Affymetrix, Inc. (Santa Clara, CA). Raw data from Affymetrix HG-U133 plus 2.0 Gene Chip microarray were normalized using the GeneChip Robust Multiarray Averaging (GCRMA) method and by removing probe sets with "absence" call on the array. The data were further calibrated by False Discovery Rate (FDR) correction with a p-value ≤ 0.05. The gene profiling data were further sub-grouped according to the JNK1 activation status in the HCC tissue relative to the ANC tissue to generate gene lists for both the high JNK1 (H-JNK1) HCC tissue and low JNK1 (L-JNK1) HCC tissue. A list of genes with a larger than 5-fold differential expression between H-JNK1 HCC and L-JNK1 HCC was also made based on direct comparison between these two groups. An online trial version of Ingenuity Pathways Analysis was used for the interactive network assay. For each gene list, independent analyses for all genes, over-expressed genes, and under-expressed genes were performed. The significance of each network was estimated as described by Lee et al.[22].

### Quantitative real-time PCR

Primers were designed using the universal probe library from Roche Applied Science (Applied Biosystems). Primer sequences and probe numbers are available upon request. Gene expression levels were analyzed by quantitative PCR in an ABI-PRISM^® ^7900 sequence detection system (Applied Biosystems) and normalized to GAPDH. Samples were run in triplicate under quantitative PCR conditions. Data were quantified and normalized using the ΔCt method. Cycling conditions PCR were 2 min at 50°C, 10 min at 95°C, then 40 cycles of 15 s at 95°C, and 1 min at 60°C.

### Analysis of patient survival data and statistics

Patient survival data were collected through the Qidong Cancer Registration System, a comprehensive cancer surveillance program, and follow-up monitoring of the patients after tumor resection. Patient survival time in months was calculated from the date of the first diagnosis of the tumor to the date of patient death. For the patients who were alive at the time of final data collection on September 30, 2008, the survival time was determined from the date of the first diagnosis to September 30, 2008. The Kaplan-Meier method was used to determine the survival probability of the patients with H-JNK1 HCC or L-JNK1 HCC. SigmaPlot 9.0 statistics software or Fisher's Exact Test was used to analyze statistic significance or clinical features.

Raw data of microarray can be found from: 

## Competing interests

The authors declare that they have no competing interests.

## Authors' contributions

QC carried out the microarray experiment and immunoblotting. JC collected HCC samples and performed statistic analysis on patient survival. KJB, VC and XS participated in the design of the study and performed bioinformatics analyses. FC designed and coordinated this study and drafted the manuscript. All authors read and approved the final manuscript.

## Supplementary Material

Additional file 1 Differentially expressed genes with FDR correction between HCC and the case-matched noncancerous liver tissues; Click here for file

Additional file 2 Differentially expressed genes between H-JNK1 HCC and the case-matched noncancerous liver tissues; Click here for file

Additional file 3 Differentially expressed genes between L-JNK1 HCC and the case-matched noncancerous liver tissues; Click here for file

Additional file 4 Commonly up-regulated genes between H- and L-JNK1 HCCs; Click here for file

Additional file 5 Commonly down-regulated genes between H- and L-JNK1 HCCs; Click here for file

Additional file 6 List of genes that are oppositely expressed between H- and L-JNK1 HCCs relative to their noncancerous liver tissues, respectively; Click here for file

Additional file 7 Differentially expressed genes between H- and L-JNK1 HCCs; Click here for file

Additional file 8 Top 5 up- and down-regulated gene pathways in H-JNK1 HCC; Click here for file

Additional files 9 Levels of HB signature genes. Click here for file

Additional files 10 Progenitor/imprinted genes (file 10) in the H-JNK1 HCC. Click here for file
