# Supplementary material for: JNK1 activation predicts the prognostic outcome of the human hepatocellular carcinoma
Source: Mol Cancer. 2009 Aug 17;8:64. doi: 10.1186/1476-4598-8-64 (PMC2732591; doi:10.1186/1476-4598-8-64)
Supplement: Additional file 8 — Top 5 up- and down-regulated gene pathways in H-JNK1 HCC; [file 1476-4598-8-64-S8.pdf]

Supplementary table 8: Top 5 pathways of the up- and down-regulated genes in H-JNK1 HCC from Ingenuity Pathway Analysis

Pathways	P-value	Molecules
**Up**		
Cellular growth & proliferation	5.71E-30-1.17E-03	226
Cell cycle	3.29E-24-1.20E-03	124
Cell Death	5.40E-23-1.16E-03	188
Cellular assembly & organization	7.21E-13-1.09E-03	62
DNA replication, Recombination	7.21E-13-1.13E-03	83
**Down**		
Lipid metabolism	6.37E-39-9.31E-04	164
Small molecular biochemistry	6.37E-39-1.19E-03	239
Amino acid metabolism	5.33E-25-1.19E-03	58
Molecular transport	7.32E-21-8.66E-04	123
Drug metabolism	1.00E-13-9.59E-04	45
