# Supplementary material for: JNK1 activation predicts the prognostic outcome of the human hepatocellular carcinoma
Source: Mol Cancer. 2009 Aug 17;8:64. doi: 10.1186/1476-4598-8-64 (PMC2732591; doi:10.1186/1476-4598-8-64)
Supplement: Additional files 9 — Levels of HB signature genes. [file 1476-4598-8-64-S9.pdf]

Supplementary Table 9:
Levels of HB signature genes in H-JNK1 HCC tissue

16 HB Signature genes	Folds of H-JNK1/L-JNK1
GHR	-19.49
APCS	-9.44
ALDH2	-9.12
C1S	-41.83
AQP9	-684.5
CYP2E1	-243.6
APOC4	-109.7
HPD	-1864
NLE	1.1
RPL10A	1.92
E2F5	1.66
BUB1	6.7
DLG7	13.2
IGSF1	1.34
AFP	53.6
DUSP9	7.7
