# Supplementary material for: JNK1 activation predicts the prognostic outcome of the human hepatocellular carcinoma
Source: Mol Cancer. 2009 Aug 17;8:64. doi: 10.1186/1476-4598-8-64 (PMC2732591; doi:10.1186/1476-4598-8-64)
Supplement: Additional files 10 — Progenitor/imprinted genes (file 10) in the H-JNK1 HCC. [file 1476-4598-8-64-S10.pdf]

Supplementary Table 10:
Expression of the progenitor and imprinted genes in H-JNK1 HCC tissue

Progenitor		Imprinted	
Genes	Folds	Genes	Folds
AFP	53.6	PEG10	206.0
KRT19	34.0	H19	65.0
TACSTD1	30.0	SGCE	29.0
DKK1	7.5	DLK1	17.7
PROM1	6.7	PHLDA2	14.0
KRT7	5.7	IGF2	12.8
THY1	2.3	PPP1AR9A	12.8
VIM	2.0	CDKNIC	11.3
		MEG3	8.5
		ZNF331	5.1
		NDN	4.9
		PEG3	4.0
		INPP5F	3.5
		SLC22A3	3.2
		ATP10A	2.5
		KCNQ10T1	2.0
		UBE3A	2.0
